# Hydroxyapatite in Oral Care Products—A Review

**DOI:** 10.3390/ma14174865

**Published:** 2021-08-27

**Authors:** Lijie Chen, Suma Al-Bayatee, Zohaib Khurshid, Amin Shavandi, Paul Brunton, Jithendra Ratnayake

**Affiliations:** 1 Department of Oral Science, Faculty of Dentistry, University of Otago, 310 Great King Street, Dunedin 9016, New Zealand; chean843@student.otago.ac.nz (L.C.); albsu711@student.otago.ac.nz (S.A.-B.); z.khurshid@postgrad.otago.ac.nz (Z.K.); paul.brunton@Otago.ac.nz (P.B.); 2 Department of Prosthodontics and Dental Implantology, College of Dentistry, King Faisal University, Al-Ahsa 31982, Saudi Arabia; 3 BioMatter Unit—École Polytechnique de Bruxelles, Université Libre de Bruxelles (ULB), Avenue F.D. Roosevelt, 50—CP 165/61, 1050 Brussels, Belgium; Amin.Shavandi@ulb.be

**Keywords:** hydroxyapatite, hydroxyapatite toothpaste, remineralisation, teeth whitening, biofilm, dentine hypersensitivity

## Abstract

Calcium phosphate compounds form the inorganic phases of our mineralised tissues such as bone and teeth, playing an important role in hard tissue engineering and regenerative medicine. In dentistry and oral care products, hydroxyapatite (HA) is a stable and biocompatible calcium phosphate with low solubility being used for various applications such as tooth remineralisation, reduction of tooth sensitivity, oral biofilm control, and tooth whitening. Clinical data on these products is limited with varied results; additionally, the effectiveness of these apatite compounds versus fluoride, which has conventionally been used in toothpaste, has not been established. Therefore, this review critically evaluates current research on HA oral care, and discusses the role and mechanism of HA in remineralisation of both enamel and dentine and for suppressing dentine sensitivity. Furthermore, we position HA’s role in biofilm management and highlight the role of HA in dental applications by summarising the recent achievement and providing an overview of commercialised HA dental products. The review also indicates the existing limitations and provides direction for future research and commercialisation of apatite-based oral care products.

## 1. Introduction

Dental caries is a global disease affecting all ages and sectors of the population. Dental caries remains the most common chronic bacterial driven disease despite advancements in early detection and treatment [1]. According to a study by *The Global Burden of Disease in 2017*, it is estimated that 2.3 billion people suffer from caries of permanent teeth and more than 530 million children suffer from caries of primary teeth [2]. Untreated caries can progress into the tooth pulp, lead to dental abscesses, cause significant pain and suffering, and ultimately tooth loss [1]. Dental caries is caused by the action of acids on the enamel surface. The acids are produced as a byproduct from bacteria in the dental biofilm (plaque) metabolising the sugars in foods or drinks [1]. The produced acid leads to a loss of calcium and phosphate from the enamel; this process is called demineralization [1]. The gingiva can also become inflamed in response to plaque irritation, diagnosed as gingivitis [3]. *The Global Burden of Disease Study 2017* also reported that the prevalence of severe periodontal (gum) diseases affect almost 10% of the global population [2]. Consequently, controlling oral microbial biofilms on the tooth surface is essential to prevent the progression of caries and periodontal disease.

Hydroxyapatite (HA) is a bioactive and non-toxic ceramic that has a close analogy to the inorganic portion of human teeth and bone. It is arranged in a typical lattice structure with the chemical formulation of (Ca_10_(PO_4_)_6_(OH)_2_) [4]. Depending on the manufacturing technique employed, various synthetic apatites are produced today [5]. HA used for biomedical applications is chemically prepared to achieve tailored properties such as chemical purity, crystal morphology, and crystal size [5]. HA’s bioactive, non-toxic, and osteoconductive properties mean it can form direct chemical bonds with living tissues. Thus, HA is a bioceramic that is widely used as an implantable material in dentistry, maxillofacial and orthopaedic surgery to repair bone defects and as a coating material for metallic implants [6]. Studies of the osteogenesis effectiveness of HA-based coatings have shown favourable results, however, their validity has been critically considered [7]. Most of these favourable results were observed in in vitro studies rather than clinical and in vivo studies, and there is a demand for more standardised comparison criteria of the published research to draw sound clinical conclusions [7]. It has also been noted that the clinical effectiveness is dependent on the specific geometry, pore size, and degradation rate of the HA, whereby a smaller particle size is desirable [8].

The biocompatibility of nano-HA has also made it attractive as a potential novel reinforcing filler in composite restorations [9]. However, so far, nano-HA has been shown to be clinically unsuitable due to high water uptake, high refractive index and hence light scattering [9]. Therefore, no significantly improved properties compared to conventional composites with regard to tooth mechanical properties and biofilm protection [9]. However, there is suggestive more promise with smaller HA particles and increasing total filler amount [9].

The external layer of human teeth, the enamel, is composed of 97% inorganic component, and the dentine is composed of 70% inorganic component; these inorganic phases are mainly made up of HA [10,11]. In the past 50 years, calcium phosphate for daily oral care has been thoroughly researched, especially in preventative dentistry. HA can be extracted from various natural resources such as bovine, ovine, porcine bone and marine structures [12,13,14,15,16]. Trace elements such as zinc, sodium, magnesium and carbonate present in some natural sources of HA have been found to mimic the apatite produced from human bone and influence the physical properties of HA such as crystal structure, morphology and solubility, and also the thermal stability [4]. Naturally sourced HA can also be a more environmentally friendly, sustainable, and economical substitute than synthetic HA [12]. However, using naturally sourced HA in oral care products has not been investigated.

HA dentifrice with synthetic HA for teeth brushing was first produced by NASA (U.S. National Aeronautics and Space Authority) as a repairing material for the astronauts who lost minerals in their teeth and bones due to the absence of gravity in 1970. In 1978, the Japanese company Sangi Co. Ltd. (Tokyo, Japan) developed the world’s first enamel restorative dentifrice. In 1993, nano-HA was approved as an anti-caries agent. Since then, in 2003, nano-HA particle size has been reduced from 100 to 50 nanometers, making it more effective at penetrating below the surface of the enamel [11,17]. Moreover, HA has also been incorporated into oral rinses and gels for oral home care to aid remineralisation and biofilm control [18,19,20].

The ideal properties of a dentifrice include minimal abrasive effect, non-irritating, non-toxic, non-staining, protects against caries and biofilm formation, while being cost-effective and readily available [21]. Many studies have been conducted to test the efficacy of HA, especially in enamel and dentine remineralisation, biofilm control, reducing dentine sensitivity, and tooth whitening [5,10,11,22,23,24,25,26,27,28,29,30]. Currently, only a few reviews report on nanomaterial in oral care products or HA in dentistry which includes restorative, preventative, and regenerative applications [5,10,11,31,32]. Therefore, this review aims to summarise current knowledge and provide an update on the claims of HA oral care products and recognise the limitations and future directions to guide clinical decision-making.

## 2. Hydroxyapatite in Oral Care Products

As HA is naturally found in enamel and dentine, synthetic HA is incorporated into dental products in various examples such as dental cement, fillings, and oral care products, including dentifrice, mouthwash, and gels [8]. HA can be synthesised in different crystallite morphologies such as spherical or needle-like and particle sizes (micro to nano) [5,33]. Currently, there are two types of HA; the nanocrystalline and the microcluster form, both nano and micro-HA are available in oral care products [10,30]. The inorganic component of enamel is made of HA crystallites in the range of ~50 nm in diameter [34]. HA crystallites are tightly packed and strictly organised as enamel prisms [5]. Micro-HA particles are about 5–10 microns in size, and this is significantly larger than enamel HA and dentinal tubules. Therefore these are not as effective in remineralisation and in reducing sensitivity [5].

**Figure 1:**
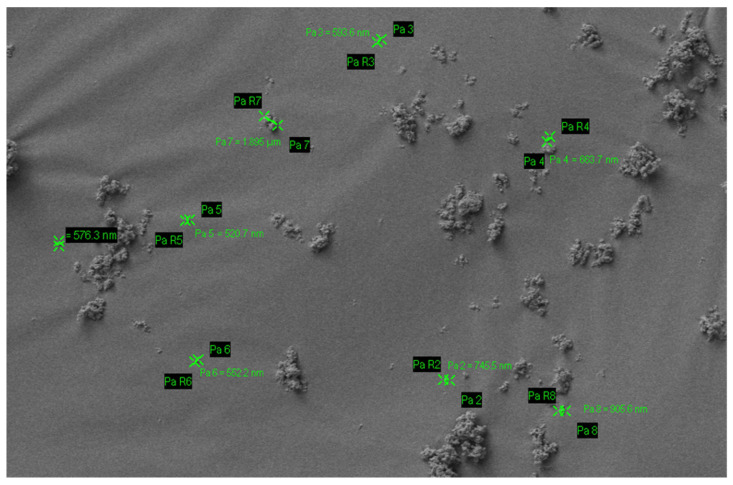
Particle size of nano-hydroxyapatite.

Nano-HA particle size ranges from 20–100 nm, with a rod-shaped morphology resembling those in natural enamel (Figure 1) [11,22,35]. Nano-HA have a high affinity to bind to substances due to an increased surface area which may improve remineralisation and reduce sensitivity as HA with a 20–50 nm size matches the nano-sized defects due to acidic erosion at the enamel surface [32,36]. Nano-HA are also thought to be more effective than micro-HA in biofilm management. The interaction with microorganisms is only possible if the particles involved are smaller than the microorganisms. Nano-size particles are small enough to directly interact with the bacterial membrane [19,32].

There are currently many toothpastes available in the market claiming to prevent dental caries, gum disease, desensitisation, tooth whitening, and remineralisation of dental hard tissues such as dentine and enamel. The anti-dental caries properties of these kinds of toothpaste are primarily based on potassium nitrates, triclosan, stannous chloride, zinc salt and fluoride compounds such as sodium fluoride, sodium monofluorophosphate, stannous fluoride. For the past few years, HA-based toothpastes have attracted more interest in the market and from manufacturers due to their biocompatibility with hard tissue and substituting capability [37,38,39]. Table 1 summarises the description of commercially available HA-based toothpastes. However, these products are still not as readily available as other products due to higher costs and limited clinical studies.

## 3. Benefits of Hydroxyapatite Dentifrice/Oral Care Products

### 3.1. Tooth Remineralisation

**Figure 2:**
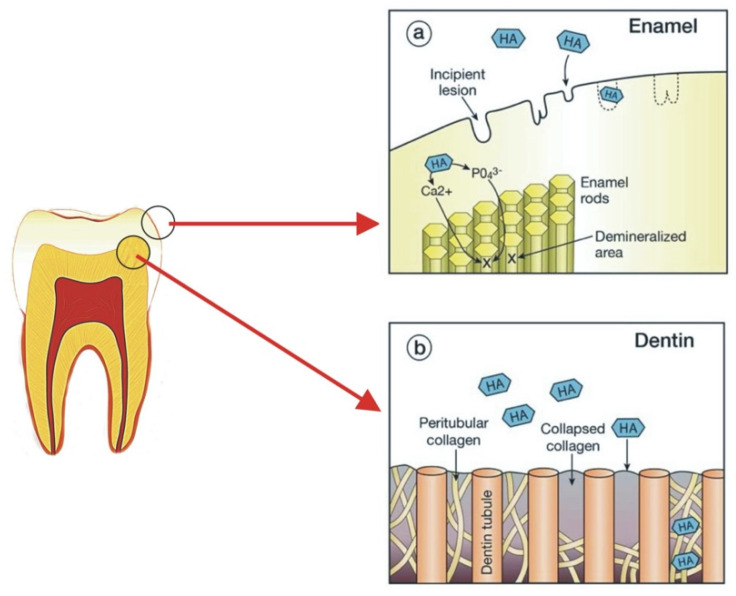
The mechanism of action of HA on the remineralisation of enamel (**a**) and dentine (**b**). In enamel, HA penetrates into porosities to replace dissolved phosphate and calcium ions and forms a layer of synthetic enamel on the tooth surface. In dentine, nano-HA penetrates into the demineralised collagen matrix acting as a scaffold for remineralisation and provides a good source of calcium locally.

**Figure 3:**
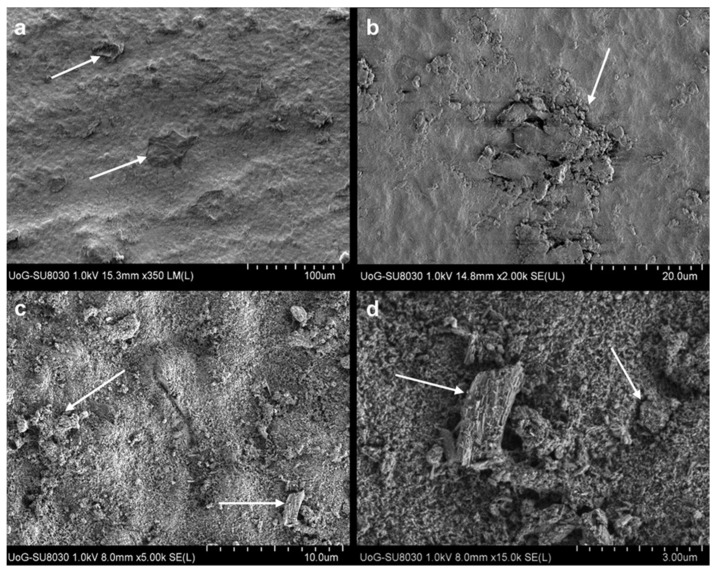
Scanning electron microscopy image shows surface HA deposits are seen on artificially demineralised enamel surfaces after treatment with Mirasensitive HA toothpaste. Subfigures a b c d shows porous enamel at increasing magnifications; (**a**) at ×350 magnification, (**b**) at ×2000 magnification, (**c**) at ×5000 magnification, and (**d**) at ×15,000 magnification. Irregularities in the enamel are filled with HA deposits (some deposits are pointed to by white arrows). Reproduced with permission from (Elizabeta S. Gjorgievska, Cambridge University Press, 2013) [46].

Modern dentistry has taken a more preventative approach through the understanding of enamel remineralisation in early carious activity [17]. Demineralised enamel and dentine have mineral ions removed from the HA crystals [40]. If the HA is partially demineralised, then this process is reversible if exposed to favourable oral environments [40]. This process is called remineralisation, where lost mineral ions in the HA crystals are restored [40]. There are many methods of remineralisation, saliva can aid in remineralisation by providing a constant source of calcium and phosphate required for the process to occur [40,41]. Fluoride also promotes remineralisation by forming fluorapatite with the calcium and phosphate released from demineralisation, this is more resistant to acid attack [40,41]. Casein phosphopeptide–amorphous calcium phosphate (CPP-ACP) is another system that promotes the precipitation of minerals to hard tooth tissue [41]. At present, there has been an increasing trend for biomimetic nano-HA to be used as a preventative agent for oral care products [17]. Research has shown nano-HA acts by providing a calcium and phosphate reservoir to remineralise enamel and dentine [8]. In dentine, nano-HA penetrates the demineralised collagen matrix, acting as a scaffold for remineralisation and providing a calcium and phosphate source locally [8]. In incipient enamel lesions, nano-HA penetrates into porosities to replace dissolved phosphate and calcium ions and forms a synthetic enamel layer on the tooth surface which can acts as a “sacrificial layer” during future acid attacks (Figure 2 and Figure 3) [8,10,11,31,42]. As when demineralisation occurs once the HA layer is deposited on the tooth surface, the dissolution of HA will produce calcium and phosphate ions which may act as a buffering solution to neutralise acids [31]. Released calcium and phosphate ions in excess can also bring the pH balance back to favour remineralisation [31].

Nano-HA has demonstrated significant remineralisation effects over micro-HA due to its smaller particle size being able to remineralise nano-sized enamel defects, and this has become the focus of HA research [29,32,43]. Table 2 provides a summary of the studies conducted on HA and its effects on remineralisation.

Several in vitro studies have proved the superior remineralisation properties of HA and fluoride; however, most studies could not prove a statistically significant difference between HA and fluoride’s remineralisation properties [18,38,43,44,45]. An in vitro study comparing the remineralisation effect between nano-HA paste and fluoride varnish on young permanent teeth concluded no significant difference in remineralisation between nano-HA and the fluoride group [44]. However, high fluoride exposure was not recommended due to the risk of ingestion in children and pregnant females [44]. Therefore, nano-HA may still be recommended as an alternative remineralising agent [44]. Likewise, similar findings were obtained in an in-vitro study that compared the remineralisation abilities between HA and fluoride gel on bovine teeth, with artificial saliva as the control [18]. Both studies used various pH-cyclical conditions to mimic acidic challenges in the oral cavity [18,44]. Alternatively, another study assessed the remineralisation effects through Vickers Hardness Number values and scanning electron microscopy (SEM) and did not find significant differences between nano-HA and fluoride [45]. Additionally, no significant changes were observed when the synergistic remineralisation effects of fluoride and nano-HA were studied [45]. Huang et al., investigated the remineralisation effects of different concentrations of nano-HA on bovine teeth. The results showed a self-limiting response where following a statistically positive correlation, the plateau was seen after nine days of cycling [43]. Thus, proving that the remineralisation abilities of HA may be effective up to a certain point [43]. Other research supports this claim and suggests that a concentration of 10% nano-HA may be optimal for remineralisation of early carious lesions [42]. The plateau is explained as an unavoidable aggregation of the particles at higher concentrations, blocking surface pores and restricting further penetration of HA into the lesion to allow for a further remineralisation effect [43]. Only one in vitro study concluded that nano-HA toothpaste had a higher remineralising effect than the amine fluoride toothpaste control on both bovine enamel and dentine [38]. However, this study did not take all oral factors into account, especially the complexity of the tooth-pellicle–plaque-saliva interface which may have impacted the results obtained [38].

In situ studies carried out provides further insight into the remineralisation effects of HA in intra-oral, biological conditions. A study was conducted to investigate the effect of different concentrations of HA or fluoride toothpaste on enamel specimens housed in the mouth of participants [17]. Results showed that all dentifrices proved effective in reducing mineral loss and lesion depth but showed no significant differences between each other in percent mineral gain [17]. These remineralisation effects are contrary to the favourable results seen in the in vitro studies. Results may be attributed to the intra-oral exposure of biological variables such as intra-oral clearance of the product, lack of particle aggregation due to saliva, and dietary differences among subjects [17]. However, another study showed that both 10% HA and 500 ppm fluoride toothpaste achieved equally significant remineralisation and reduction in lesion depth [30]. Thus, showing promising results for HA in remineralisation. In recent years, few clinical studies on the remineralisation of HA have been carried out [23,24]. The most popular modality of assessing HA remineralisation effects was comparing carious lesion prevalence before and after HA intervention [28]. A randomised control trial (RCT) by Schlagenhauf et al., investigated the effect of nano-HA dentifrice in comparison with fluoride dentifrice (1400 ppm) on vestibular caries in orthodontic patients [23]. The results were comparable for nano-HA and fluoride, and they concluded there was no clinical superiority of nano-HA dentifrice over fluoride dentifrice [23]. In contrast, a RCT conducted in children showed that a commercial micro-HA toothpaste (Karex™) was clinically non-inferior to a fluoride toothpaste in preventing primary teeth enamel lesion progression [24]. Due to the biomimetic nature of HA unlike fluorides (e.g., risk of dental fluorosis), HA is biocompatible and is safe if accidentally swallowed [31]. Therefore, HA-based toothpaste could be useful in the prevention of early childhood caries (ECC) and for bedridden patients who are compromised due to disabilities [24]. In conclusion from the above studies, the remineralisation effects of nano-HA have been proven to be equal to fluoride [23,24,30,38,43,44]. Nevertheless, its biomimetic and non-toxic properties are a benefit over fluoride.

Natural enamel and dentine consist of HA with trace amounts of other ions such as Na^+^, Mg^2+^, Fe^2+^, F^−^, Zn^2+^ [36]. To mimic the natural composition of human teeth and bone there are several studies investigating the addition of ionic substitutes into synthetic HA [4,36]. Substitutions in the HA lattice can change the physical properties of HA, including crystal structure, morphology, solubility, thermal stability, and bioactivity [36]. The remineralisation abilities of HA can be improved by substituting ions such as zinc, fluoride, magnesium, and strontium to the HA lattice [47]. The addition of these ions improves the stability, solubility, and strength of the HA, aiding in the remineralisation potential of enamel surface lesions and in preventing biofilm formation [47]. Beta-tricalcium phosphate (β-TCP) is another attractive calcium phosphate system as it can emerge as a transitional phase in the conversion of HA. Furthermore, it is compatible with biological systems and is bioactive [4,36]. In a study where the β-TCP structure was altered by combining with carboxylic acids and surfactants to generate functionalised β-TCP (fTCP), the toothpaste containing fTCP and fluoride increased remineralisation of the artificial enamel subsurface lesions during pH-cycling [48]. In another study, fTCP containing toothpaste effectively reduced white spot lesions (WSL) compared to 1000-ppm fluoride-containing toothpaste [49]. However, an in situ study showed that the remineralisation efficacy of fTCP toothpaste was similar to that of fluoride toothpaste [50]. Although β-TCP has shown the potential for tooth remineralisation, further investigations are required to establish its clinical efficacy.

Currently, novel research has also been interested in investigating the synergistic effects of fluoride and nano-HA. A study investigating sodium fluoride (NaF) combined with HA demonstrated a synergistic effect between NaF and nano-HA for remineralisation [51]. However, more studies are needed to determine the optimal nano-HA and NaF mouth rinse conditions for human use [51]. This is supported by a study that investigated the synergistic effects of fluoride and nano-HA on preventing enamel demineralisation around orthodontic brackets [29]. Results revealed significantly less demineralisation depth in the combined nano-HA and fluoride group [29]. Additionally, the combination of nano-HA with fluoride has shown better enamel resistance to erosion in bovine teeth [52]. Meyer et al., concluded that the addition of fluorides to calcium phosphate may reduce the bioavailability of fluoride intraorally by forming insoluble compounds, including calcium fluoride or fluorapatite [10]. As data in this area are scarce, future investigations are required to gauge whether there are any benefits in incorporating both fluoride and HA in oral care products. The synergistic effect of HA with other substances or therapy is also of interest. A RCT investigated the remineralisation between nano-HA gel, in-office ozone therapy, and a combination of both compounds [28]. Results showed that a higher rate of remineralisation was seen when nano HA and ozone therapy were used in conjunction [28]. Furthermore, when the combined effects of Galla Chinensis (GCE: a traditional Chinese medicine) with nano-HA was investigated, results proved a significant synergistic effect of combined GCE and nano-HA on the depth reduction of initial enamel lesions [53]. More research is required on the mechanism of this synergistic effect. However, these studies give promising insight and research possibilities of investigating other substances and therapy when used together with HA oral care products.

### 3.2. Dentine Hypersensitivity

**Figure 4:**
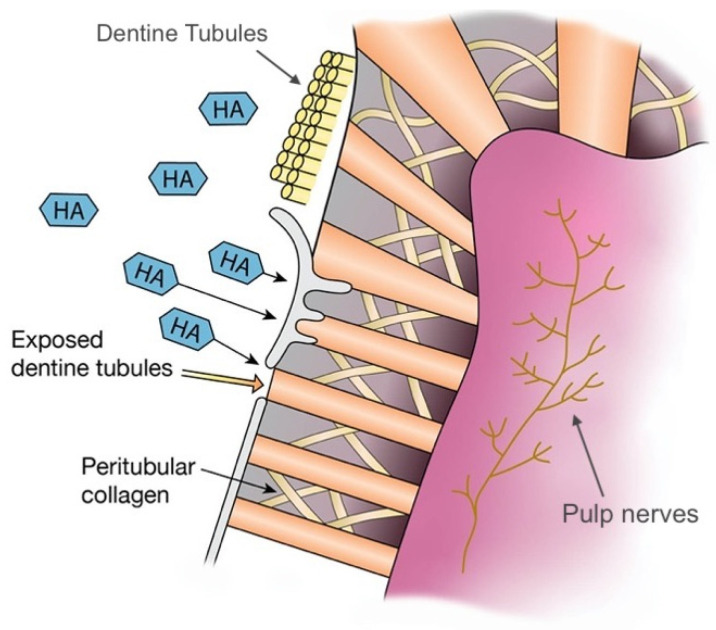
The mechanism of action of HA on the management of dentine hypersensitivity. HA can occlude the exposed dentinal tubules.

Dentine hypersensitivity is one of the most prevalent dental conditions, affecting 8–57% of the adult population [54]. Its occurrence has been explained by a few theories, with the most favoured being the hydrodynamic theory [55]. The hydrodynamic theory explains that open dentinal tubules allow fluid movement, which stimulates the pulp nerves to induce pain [55]. Various kinds of toothpaste have been developed to reduce tooth hypersensitivity, where their components occlude dentinal tubules to reduce hydraulic conductance and minimise sensitivity [55]. The effect is enhanced by their remineralisation abilities, where a mineralised layer creates a barrier [55]. Dentifrices containing potassium ions can also depolarise pulpal sensory nerves and interrupt the transmission of pain stimuli [55]. Nano-HA has been proposed to combat tooth sensitivity more effectively compared to conventional toothpaste by bio-chemically binding to both collagen and HA from dentine, and due to their nano-sized diameters, they are able to reliably occlude the tubules (Figure 4) [31]. Studies that investigate the effect of HA on teeth sensitivity are shown in Table 2.

The majority of studies investigating the effectiveness of nano-HA on tooth sensitivity are based on clinical studies due to the subjective nature of tooth sensitivity. In various comparative clinical investigations, nano-HA has proven superior over controls such as fluoride and potassium nitrate in reducing tooth hypersensitivity [55,56,57]. In a RCT, a commercial nano-HA toothpaste (PrevDent^®^) showed reduced tooth sensitivity compared to fluoride toothpaste [55]. A similar effect was seen when HA was compared against a placebo toothpaste, suggesting that HA is more effective in occluding dentinal tubules compared to the controls [56,57]. These two studies minimised bias through the placebo effect by having a placebo control group [56,57]. When the effectiveness of zinc-carbonate hydroxyapatite (Zn-CHA) nanocrystals dentifrice was compared with potassium nitrate/fluoride dentifrice, Zn-CHA proved superior once again [56]. In this study, Zn-CHA dentifrice is suggested to work through a dentinal tubule plugging action, and potassium nitrate through depolarising pulpal nerve action [56]. An explanation for the reduced dentine hypersensitivity in the test dentifrice was its lower abrasiveness when compared to that of the control dentifrice [56].

Various non-comparative, quasi-experimental clinical studies have also been carried out on the topic. A study was conducted to evaluate the effectiveness of a desensitising toothpaste containing potassium nitrate, sodium monoflurophosphate, and nano-HA, as well as antioxidants phloretin, ferulic acid, and silymarin [58]. The dentifrice showed significant improvement in sensitivity after 48 h and two weeks, highlighting its effective speed in relieving dentine hypersensitivity [58]. A similar study that used a toothpaste containing Zn-CHA nanocrystals over a more extended period of four to eight weeks, found statistically significant reductions in tooth sensitivity, highlighting Zn-CHA toothpaste’s long-term clinical effects [59]. However, a major limitation of these studies is that they do not have placebo controls, with each subject being their control [58,60]. In vitro studies on the effects of HA on tooth sensitivity have also been conducted. These provide quantification of tubule occlusion and statistical data through the assessment of SEM images [54]. An in vitro study was conducted to evaluate and compare the effects of three different desensitising denitrifies; a nano-HA containing dentifrice, Novamin (commercially available bioactive glass-based toothpaste that consists of calcium sodium phosphosilicate), and Proargin (a system that contains both calcium carbonate and arginine) on dentinal permeability and tubule occlusion [54]. Results showed a higher percentage of dentinal occlusion with nano-HA when compared to Proargin and Novamin [54]. However, these findings are inconsistent with those of Dhillon et al., which showed the superiority of calcium sodium phosphosilicate over Proargin and HA [61]. The differences may be due to processing differences in the dentin specimens and different regimes of desensitising dentifrice application [54]. A combined in vitro and in vivo study highlighted the importance of considering the in vivo effects in comparison to the in vitro effects as they can produce contradictory results [62]. As when their study investigated the in vitro effects in the absence of saliva, HA containing toothpaste was most effective in reducing dentine permeability, and arginine and calcium carbonate was the least [62]. In contrast, in the presence of saliva and ageing, HA dentifrice was the least effective, and arginine and calcium carbonate were the most effective at reducing dentine permeability [62].

From the reviewed literature, it can be concluded that HA dentifrices have shown definite clinical efficacy in reducing dentine hypersensitivity both in the immediate and long term [58,59]. However, the placebo effect remains a major limitation in investigating tooth sensitivity [58,60]. Many comparative in vitro studies exist between desensitising toothpaste, but further investigations of their in-vivo implications are required [62]. Additionally, many quasi-experimental but not enough comparative clinical studies were conducted between existing desensitising toothpaste [58]. Continued research is required in this area to highlight the beneficial plausibility of nano-HA dentifrice over already existing desensitising toothpastes.

### 3.3. Oral Biofilm Management: Effect on Periodontal Conditions

In patients with periodontal and gingival disease, as well as mechanical removal of plaque, the addition of pharmacological agents to biofilm control has also been an area of focus in recent years, which may support favourable treatment outcomes [63]. The development of biofilm control using fluoride has effectively reduced plaque formation and improved periodontal health, leading to fluoride being the gold standard in oral care products [63]. Likewise, chlorhexidine (CHX) as an antimicrobial agent is the gold standard in a mouth rinse. However, current issues include long-term use of CHX, resulting in side effects such as tooth discolouration and dysbiosis of oral ecology [64]. HA‘s role in biofilm control has been an area of interest recently due to its biomimetic properties in keeping the microbiome in balance [64].

**Figure 5:**
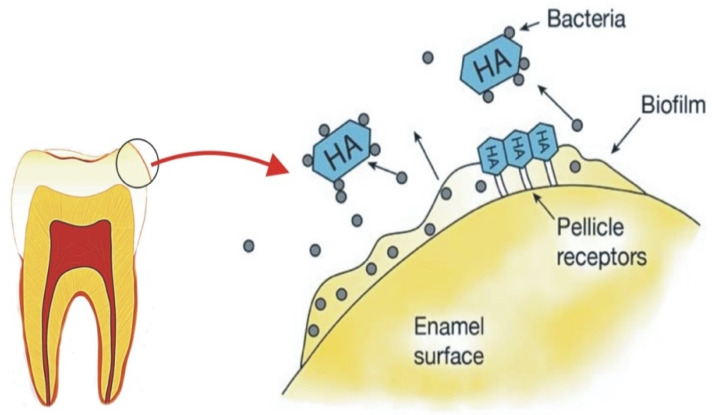
The mechanism of action of HA on biofilm management. HA can bind to microorganisms by interacting with the bacterial adhesin thus agglutinating bacteria and removing them from the oral cavity. HA also blocks the pellicle receptors from binding to bacterial receptors.

HA is effective in biofilm control through mainly an anti-adhesive property by preventing bacteria from binding to the tooth surface [11,63,64,65]. The mechanism of action (Figure 5) is explained by the binding of microorganisms to free HA particles incorporated into dentifrice or mouth rinse [10,31]. HA also binds to proteins and plaque; therefore, it inhibits the development and aggregation of biofilm on the enamel surface [11,63,64]. HA particle size is important as nano-size HA exhibits greater active surface area, which influences the stability of incorporation into the biofilm and interaction with the bacteria cell membrane [19,65]. Table 2 provides a summary of studies that investigated the effect of HA on biofilm control.

A couple of in situ studies by Kensche et al. and Hannig et al., investigated the impact of pure HA microcluster on the initial bacterial colonisation at the enamel surface. It was concluded that HA particles in mouth rinse could prevent biofilm formation, and the effect is comparable to CHX [19,65]. However, the mechanism differs from CHX as HA acts as an anti-adhesive rather than an antibacterial agent [65]. Results from Kensche et al., also suggested two mechanisms that HA can achieve the inhibition of biofilm formation. One mechanism suggested that the HA microcluster block the pellicle receptors from binding to bacterial receptors. The other mechanism suggested that HA microcluster can interact with the bacterial adhesin, thus agglutinating bacteria and removing them from the oral cavity [65]. A mouth rinse with Zn incorporated HA exhibited anti-adherent and antibacterial effects [19]. However, the antibacterial effect can be explained by the adhesion of Zn into the HA [19].

An in vivo study was conducted to analyse the quantitative parameters of micro-HA in improving periodontal disease in patients [63]. The study concluded that HA toothpaste had comparable effects to amine and stannous fluoride toothpaste in reducing plaque index, bleeding on probing, and the gingival index, thus improving periodontal health [63]. Another in vivo study drew a similar conclusion where HA mouth rinse was proven to be as effective as CHX in plaque reduction and gingival index [66]. In another study that used questionnaires to evaluate the effect of HA toothpaste on various parameters, results showed that a subjective feeling of “tooth smoothness” was reported by participants [25]. Such results can be explained by the reduction of bacterial colonisation and biofilm formation due to binding to HA particles [25].

Substituting ions into HA not only allows synthetic HA to be more biomimetic, but it also improves properties such as the antimicrobial ability of HA. Zinc and silver have been extensively researched in this area, with results showing that exposure to zinc substituted HA reduced the bactericidal viability of *Streptococcus mutans* [19,63]. It has been proven that zinc ions can inhibit bacterial metabolism and prevent halitosis by inhibiting volatile sulphur compounds [10]. Research on the antimicrobial properties of silver has also been long established [36]. Studies have shown that silver ions leached out of the HA structure can inhibit the growth of *Escherichia coli*, *Staphylococcus aureus*, and *Pneumococcus* [36]. The substitution of other cations and anions has been extensively researched; however, the focus has been more on implants and bone regeneration [36]. More evidence is needed for their benefits in oral care before these ion-substituted HA can be added to oral care products promisingly.

In summary, in situ and in vivo studies have found that the effect of HA on biofilm formation on the tooth surface is comparable to CHX and amine/stannous fluoride [19,63,65,66]. Therefore, it is a sensible supplement in oral care. HA particles have been found to do so without any antibacterial effect due to their anti-adherent properties [65,67]. Therefore, acting as a biomimetic biofilm control without disrupting the biofilm ecological balance in the oral cavity [19]. Currently, evidence suggests toothpaste and mouth rinse with nano and micro-HA can contribute to oral biofilm management [19,63,65,66]. More in-situ and in-vivo studies with an increased number of participants and a longer time frame need to be conducted to provide optimal insight into the mechanism of the anti-adhesive property of HA in oral care products to stimulate natural oral conditions.

### 3.4. Teeth Whitening

Age-related changes and lifestyle habits result in darker teeth colour [68]. An increasing number of oral care products has shifted the focus towards teeth whitening mainly due to cosmetic reasons. White teeth and a bright smile are preferred by many and can lead to an improved quality of life [68].

Although HA-incorporated oral care products have been proven to be effective in preventative dentistry through remineralisation, it is only in the past 20 years that research has been directed to investigate the whitening properties of HA [68]. In vivo and in vitro studies have been carried out to understand HA’s teeth whitening mechanism compared to the whitening property of conventional whitening products such as hydrogen peroxide and other commercial dentifrice and rinses [26,27,69,70,71,72,73]. Studies have also investigated the effect and efficacy of teeth whitening by HA [5]. Table 2 shows a summary of studies that investigated the whitening effects of HA oral care products. HA’s effect on whitening is physiological rather than mechanical or chemical [71]. HA increases the brightness and whiteness of the tooth surface by remineralisation which adds to the smoother and glossier appearance [31,71].

A combined experimental and clinical study investigated both the polishing properties and the brightening and whitening properties of dentifrice with three different concentrations of HA [71]. It concluded that the addition of HA to dentifrice resulted in a dose-response relationship between the concentration of HA and the brightness and whitening of the tooth surface [71]. Results also showed that the addition of HA did not alter its polishing properties; therefore, the whitening effect is not due to abrasiveness [71]. Results also show that after four months of use of HA dentifrice, the brightening and whiteness became stable due to the remineralisation effect, which turns the tooth surface smoother and glossier up to a particular state [71]. The whitening effect is explained by the adherence of HA to the tooth surface, forming a layer of synthetic enamel. As HA is white, it will reflect more light resulting in an increase in brightness for a long-lasting whitening effect [73].

In two randomised, double-blind clinical trials with 150 and 85 participants respectively, one showed that dentifrice containing HA or calcium peroxide did not produce any reduction in tooth staining compared with a placebo fluoride dentifrice [70]. The other showed that the hydrogen peroxide-containing dentifrice caused significant lightening of tooth colouration than the HA and placebo dentifrice [69]. An observational pilot study showed that the subjective whitening perception of HA dentifrice of 25 participants was increased to varying degrees over four weeks of use [26]. However, no long-term clinical data are available on the whitening properties of HA, which is a limitation [69,70,71,74].

Multiple studies have concluded that the addition of HA in dentifrice has a whitening effect on the tooth surface that is not as damaging as other methods that use abrasion or oxidation, is easier to apply as it can be self-administered, and provides a good alternative [26,27,71,73]. In addition, a whiter tooth colour was observed after one-time application when HA dentifrice was used, which could be enhanced after regular use [27,75]. However, the whitening degree may only be equal to 50% of the whitening effect of bleaches like hydrogen peroxide [74].

**Table 2:** Studies conducted on the claims of HA oral care products.

Type of Study	Method	Comparison Group	Main Findings	Reference
Remineralisation				
In situ	Mineral loss and lesion depth of each specimen were quantified using microradiography	Fluoride toothpaste	All dentifrices were effective in reducing mineral loss and lesion depth but showed no significant differences between each other in percent mineral gain.	[17]
In situ	Microradiography	500 ppm fluoride toothpaste	10% HA achieved comparable efficacy with 500 ppm fluoride in remineralising initial caries and preventing demineralisation.	[30]
In vitro	%SMHR, SEM	Deionised water (negative control), NaF positive control	Nano-HA had the potential to remineralise initial enamel lesions. A concentration of 10% nano-HA may be optimal for remineralisation of early enamel caries.	[42]
In vitro	DIAGNOdent	Fluoride varnish	Significant remineralisation effects of both fluoride and nano-HA paste compared to the control group: no treatment. However, there were no statistically significant differences between the groups.	[44]
In vitro	Mineral loss was quantified using microradiography	Fluoride-based gel or artificial saliva	Both fluoride and HA showed effective remineralisation abilities but there were no statistically significant differences between the groups.	[18]
In vitro	Remineralisation effects were studied with Vickers Hardness Number and SEM image of the enamel surface	Nano-HA with fluoride	Significance in remineralisation for nano-HA with and without fluoride, however not statistically significant effects from each other.	[45]
In vitro	Remineralisation effect investigated through surface and CSMH tests and PLM	-	Nano-HA > Micro-HA.	[43]
In vitro	Differences in mineral loss evaluated with microradiography	Amine fluoride toothpaste	Nano-HA toothpaste showed higher remineralising effects compared to amine fluoride toothpastes in bovine dentine and enamel.	[38]
In vivo RCT	Remineralisation effects were investigated through comparative bitewing radiographs before and after treatment	In-office ozone therapy, or a combination of both	The smallest rate of remineralisation was seen with nano-HA treatment alone, and the highest rate was seen when nano-HA and ozone therapy were used in conjunction.	[28]
In vivo RCT	ICDAS ≥ code 1, ICDAS ≥ code 2, the plaque index, and the gingival index	Fluoride dentifrice	No statistical difference between nano-HA and fluoride dentifrice.	[23]
In vivo RCT	The development of ICDAS of more than or equal to 1	Fluoride toothpaste	Micro-HA toothpaste was clinically non-inferior compared to fluoride toothpaste in preventing primary teeth enamel lesion progression.	[24]
Dentine hypersensitivity				
In vivo RCT	Airblast, tactile tests, and VAS of pain to stimuli	Fluoride dentifrice and placebo	Statistically significant lower values of sensitivity with nano-HA compared to fluoride and placebo in subsequent weeks of use. Significantly lower values of cold air and tactile sensitivity, and VAS scores.	[55]
In vivo RCT	Airblast, tactile, cold water, and subjective tests	Potassium nitrate/fluoride dentifrice	Both CHA and potassium nitrate/fluoride dentifrice were significantly effective in reducing dentine hypersensitivity among subjects. Statistically significant improvement in airblast score and subjective scores with CHA toothpaste. In contrast, no significant difference between groups for tactile or cold-water tests.	[56]
In vitro	SEM	Novamin, Proargin, normal saline	All three were effective in dentine tubule occlusion. Statistically significant difference on increasing dentinal tubule occlusion between HA and Proargin. Although HA occluded dentinal tubules more than Novamin, findings here were not statistically significant.	[54]
In vitro	SEM	Calcium sodium phosphosilicate, Proargin, normal saline	All three were effective in dentine tubule occlusion, but calcium sodium phosphosilicate showed significantly higher tubular occlusion compared to other groups.	[61]
In vivo RCT	VAS	-	Significant improvement in sensitivity between 52–76% after 48 h, and 70–84% after two weeks.	[58]
In vivo RCT	Airblast method using Schiff Sensitivity Scale.	-	Statistically significant differences were observed between baseline and four and eight-week intervals.	[59]
In vivo RCT	Questionnaire with VAS and Likert scales	-	Biomimetic zinc HA was effective in reducing dentin hypersensitivity.	[25]
In vivo RCT	VAS and EPT	Placebo paste	Sensitivity scores significantly decreased in HA group compared to placebo group.	[57]
In vitro and In vivo	Hydraulic conductance through commercially available capillary flow system. Once without saliva (in-vitro) and once with saliva and ageing to replicate biological conditions (in-vivo)	Potassium nitrate, and an arginine and calcium carbonate-containing toothpaste	—In vitro: In the absence of saliva, HA containing toothpaste was most effective in reducing dentin permeability, and arginine and calcium carbonate was the worst —In vivo: In the presence of saliva and ageing, HA was the worst, and arginine and calcium carbonate was the best.	[62]
Biofilm management				
In situ	DAPI and live/dead staining, SEM	Chlorhexidine	HA particles reduce initial biofilm formation on enamel surface is comparable to chlorhexidine.	[65]
In situ	DAPI and live/dead staining	Chlorhexidine	Mouthwash containing HA reduces initial biofilm formation comparable to chlorhexidine.	[19]
In vivo RCT	Plaque formation rate, plaque control record, gingival index, bleeding on probing, pocket probing depth	Amine and stannous fluoride toothpaste	HA toothpaste reduced plaque index, bleeding on probing and gingival index but did not change the plaque formation rates. This is comparable to amine and stannous fluoride toothpaste.	[63]
In vivo	Plaque and gingival index, DIAGNOdent	Chlorhexidine	HA is as effective as chlorhexidine in plaque reduction.	[66]
In vivo	Questionnaire VAS and Likert scales both at baseline and follow-up	-	Subjective feeling of tooth-smoothing can be explained by the reduction of bacterial colonisation by HA particles. A stronger feeling of freshness after toothbrushing was also reported.	[25]
In vivo	Levels of calcium and phosphorus of plaque samples were analysed by energy-dispersive X-ray spectroscopy	-	HA may be incorporated into the oral biofilm and/or may adhere to dental plaque.	[76]
In vivo RCT	Paired-end Illumina Miseq 16S rDNA sequencing	AmF/SnF_2_	Toothpaste containing anti-adhesive HA did not induce statistically noticeably different changes in microbial composition compared to an antimicrobial and anti-adhesive AmF/SnF_2_ formulation.	[77]
Teeth whitening				
Combined experimental and clinical study.	Weighed using a fine balance, thickness-loss (nm) per cm^2^ per hour Two colourimeters (SZ-Y-90 and SE-2000) with two specially made fiberscopes (inner diameters of 3.5 and 2.5 mm)	-	1. Different amounts of HA in toothpaste do not change the polishing properties. 2. HA toothpaste increased teeth brightness and whiteness. Has a dose-response relationship. 3. No correlation between polishing and whitening properties of HA toothpaste.	[71]
Combined experimental and clinical study	Photoresearch Spectra-Scan PR-650 photocolorimeter SEN (Hitachi S-4500)	An identical toothpaste without HA	HA toothpaste can alter tooth colour by at least one shade with daily brushing. However, this is less powerful than harmful bleaches such as peroxides.	[74]
In vitro	Dental spectrophotometer	-	Synthetic nano-HA is an alternative for tooth whitening because they have some advantages when compared to oxidising bleaching materials. All nano and micro-HA tested exhibited whitening effects of variable degrees on the enamel surfaces.	[73]
In vitro	Colour changes (ΔE) were measured spectrophotometrically	Whitening mouth rinse with phosphates, or negative control (distilled water)	Significantly higher ad hoc whitening effect of the HA gel compared to the mouth rinse and water after short-time application.	[27]
In vivo, pilot study	Questionnaire regarding their perception of their tooth colour and brightness	-	Micro-HA is a promising whitening agent for oral care formulations and represents a biomimetic alternative to other whitening agents for daily dental care.	[26]
In vivo RCT	Vita Easyshade (Vita 3D-master scale) and Degudent Shadepilot (Classical Vitashade scale)	Calcium peroxide and no active ingredient (placebo)	Toothpaste containing HA or calcium peroxide did not produce any reduction in tooth staining compared with a placebo fluoride toothpaste.	[70]
In vivo RCT	ShadeEye NCC and Vita classical shade guide, VAS scale (range, 1–5)	Hydrogen peroxide and placebo	Hydrogen peroxide-containing toothpaste caused significant lightening of tooth colouration than the HA and placebo toothpaste.	[69]
In vitro	VITA shade scores of Shadeeye-EX NCC Dental Chroma meter (Shofu Co. Japan)	Group 1, a new toothpaste containing (Nano-HA) Group 2: toothpaste containing silica and multi phosphate. Group 3: toothpaste containing abrasives with silica and micro-sized HA	No significant differences in shade change between each group (*p* > 0.05). New Nano-HA toothpaste had similar whitening efficacy to commercially available whitening toothpaste.	[72]
Combined In vitro and in vivo study	A spectrophotometer. Proof-of-concept clinical study was performed investigating the mixture of SAPM+HA	-	The combination of SAPM+HA particles caused optical whitening based on diffuse reflection by the HA particles on the tooth surface. The whitening effect and its magnitude observed in vitro were also seen in vivo.	[75]
In vitro	Dental spectrophotometer	-	Calcium phosphate-based formulations that can adhere to the enamel surface have promising tooth-whitening potential.	[78]

HA: hydroxyapatite; SEM: scanning electron microscopy; %SMHR: Percentage surface microhardness recovery; NaF: sodium fluoride; DIAGNOdent: laser examination tool for early detection of caries; CSMH: cross-sectional microhardness; PLM: polarised light microscopy; ICDAS: International Caries Detection and Assessment System; RCT: randomised controlled trial; VAS: visual analogue scale; CHA: carbonate hydroxyapatite; Novamin: commercially available bioactive glass-based toothpaste that consists of calcium sodium phosphosilicate.; Proargin: a system that contains both calcium carbonate and arginine; EPT: electric pulp test; DAPI: fluorescent stain that binds strongly to adenine–thymine-rich regions in DNA; DLS: dynamic light scattering; AmF/SnF_2_: amine fluoride/stannous fluoride toothpaste; EDS: energy-dispersive X-ray spectrometry; MTT assay: a colourimetric assay for assessing cell metabolic activity; SAPM: self-assembling peptide matrix.

We can conclude that research on teeth whitening by HA oral care products is still novel. Nevertheless, results have aided in understanding the mechanism of HA’s mechanism in improving the whiteness and brightness of teeth. Results also reveal that all nano and micro-HA materials tested exhibit enamel whitening effects of variable degree [26,27,69,71,72]. However, no studies to date have directly compared the tooth whitening effectiveness of the two against each other. However, based on the tooth whitening mechanism explained above, it may be speculated that since nano-HA has been superior in enamel remineralisation, it is likewise for tooth whitening. Therefore, another future direction for investigation would be to evaluate the effect of particle size of HA on teeth whitening for various age groups. Thus, more in vivo studies that are well designed and with a longer time frame need to be carried out to confirm the whitening effects of HA in oral care products. The use of standardised shade guides before and after a determined and long duration use of nano-HA toothpaste could be a starting place for this.

## 4. Conclusions and Future Perspective of Hydroxyapatite Oral Care Products

There is a growing demand for a more biomimetic material in oral care products. HA’s resemblance to natural enamel and dentine has been a focus in recent years, highlighting its bioactive and non-toxic properties. Both micro and nano-HA are used in oral care products including mouth rinses and toothpastes with various claims, including remineralisation, biofilm management, dentine hypersensitivity, and teeth whitening. Research has shown comparable effectiveness for HA when compared to fluoride in remineralisation and CHX in biofilm management. HA’s effectiveness in reducing dentine hypersensitivity and whitening teeth appears to be promising too. Although the evidence for HA is comparable to other substances, its non-toxic and biomimetic property provides an advantage over conventional products. In particular, in biofilm management and teeth whitening conventional products, including CHX and peroxides, have proven side effects when used in the long term. Our understanding of HA’s properties and how they can be improved with the addition of other ions and substances are growing. Although the addition of HA to oral care products has been extensively researched, more clinical studies are required to highlight and facilitate HA’s inclusion in oral care products.

## Figures and Tables

**Table 1:** Commercial toothpaste based on hydroxyapatite (nano).

S. No	Commercial Product	Ingredients	Country
1	APAGARD^®^ PREMIO	Aqua, dicalcium phosphate, glycerin, xylitol, hydroxyapatite (nano), silica, Peg-8, sodium lauryl sulfate, cellulose gum, aroma, sodium silicate, trimagnesium phosphate, hydrolyzed conchiolin protein, sodium saccharin, glycyrrhetinic acid, cetylpyridinium chloride, lauryl diethylenediaminoglycine Hcl	Germany
2	Ela Mint Toothpaste	Water, vegetable glycerin, hydrated silica, sorbitol powder, silica, hydroxyapatite (nano), sodium benzoate, sodium lauroyl sarcosinate, mentha piperita essential (peppermint) oil, *Mentha viridis* (spearmint) oil, *Illicium verum* (star anise) oil, *Gaultheria procumberis* (wintergreen) oil, xylitol, xanthan gum, *Stevia rebaudiana* extract powder, methylsulfonylmethane, *Aloe barbadensis* (aloe vera) leaf juice, sodium bicarbonate, *Camellia sinensis* (green tea) leaf extract, *Cucumis sativus* (cucumber) fruit extract, *Persea gratissima* (avocado) fruit extract, *Mangifera indica* (mango) fruit extract, menthol, *Elettaria cardamomum miniscula* seed (cardamom), potassium chloride.	USA
3	Coco Ginger Toothpaste	Glycerin, water, hydrated silica, erythritol, silica, natural flavors (coconut and ginger), hydroxyapatite (nano), xanthan gum, sodium benzoate, *Aloe barbadensis* (aloe vera) leaf juice, *Chamomilla recutita* (chamomile) flower extract, methylsulfonylmethane (msm), potassium chloride, sodium bicarbonate, *Stevia rebaudiana* extract powder, sodium lauroyl sarcosinate.	USA
4	APAGARD^®^ RIN-SU	Aqua, glycerin, xylitol, hydroxyapatite, xanthan gum, alcohol, polyglyceryl-5 stearate, lauryl diethylenediaminoglycine HCL, aroma, cethylpyridinium chloride	Germany
5	APADENT^®^ TOTAL CARE	Aqua, dicalcium phosphate, glycerin, hydroxyapatite (nano), silica, peg-8, sodium lauryl sulphate, cellulose gum, aroma, trimagnesium phosphate, pvp, butylene glyium sodium, alcoholic acrylic, sodium sodium, sodium, sodium, sodium, sodylacride, sodylen, sodylacride, sodylacride, sodyl. pyridoxine HCL, lauryl diethylenediaminoglycine HCL, *Camellia sinensis* leaf extract, *Chamomilla recutilla* (Matricaria) extract, *Salvia officinalis* (Sage) leaf extract	Germany
6	Travel Size Ela Mint Toothpaste	Water, vegetable glycerin, hydrated silica, sorbitol powder, silica, hydroxyapatite (nano), sodium benzoate, sodium lauroyl sarcosinate, *Mentha piperita* essential (peppermint) oil, *Mentha viridis* (spearmint) oil, *Illicium verum* (star anise) oil, *Gaultheria procumberis* (wintergreen) oil, xylitol, xanthan gum, stevia rebaudiana extract powder, methylsulfonylmethane, *Aloe barbadensis* (aloe vera) leaf juice, sodium bicarbonate, camellia sinensis (green tea) leaf extract, *Cucumis sativus* (cucumber) fruit extract, *Persea gratissima* (avocado) fruit extract, *Mangifera indica* (mango) fruit extract, menthol, *Elettaria cardamomum miniscula* seed (cardamom), potassium chloride.	USA
7	Toothpaste PrevDent^®^ nHAp™	Aqua, hydrated silica, sorbitol, glycerin, xylitol, potassium nitrate, nano-hydroxyapatite, magnesium aluminum silicate, mentha piperita oil, sodium lauroyl sarcosinate, xanthan gum, phenoxyethanol, potassium chloride, sodium sulfate, sodium saccharin, CI 77891	The Netherlands
8	Biorepair^®^	Sorbitol, Aqua, Silica, PEG32, Glycerin, Aroma, Zinc hydroxyapatite, Na-Myristoyl Sarcosinate, Celulose gum, Citric acid, Na-benzoate, Benzylalcohol, Na-methyl cocoyl taurate, Mentha peprita oil, Na-sacchrine, K-sorbate, Fragaria vesca Juice, Anethole, Phenoxyethanol, Mentho	Italy
9	X-PUR Remin^®^	10% Nano Medical Hydroxyapatite 10% Xylitol, Water, macrogol 400, zeolite, polyvinylpyrrolidone, glycyrrthetinc acid, cetylpyridinium chloride, glycerin, xylitol silicic anhydride, castor oil, sodium lauroyl glutamate, carragenan, ethanol, carboxymethylcellulose sodium, titanium dioxide, flavour.	Canada
10	Biorepair^®^ Advanced Active Shield Anti-Cavities	Aqua, zinc hydroxyapatite, hydrated silica, sorbitol, glycerin, xylitol, silica, aroma, cellulose gum, zinc pca, sodium myristoyl sarcosinate, sodium methyl cocoyl taurate, tetrapotassium pyrophosphate, sodium saccharin, zinc citrate, citric acid, ammonium acryloyldimethyltaurate/VP copolymer, benzyl alcohol, phenoxyethanol, sodium benzoate, limonene.	Italy
11	GUM SensiVital+ toothpaste	Glycerin, aqua, hydrated silica, isomalt, potassium nitrates, hydroxyapatite, PVM/MA copolymer, lauryl glucoside, PEG-40 hydrogenated castor oil, sodium monofluorophosphate, aroma, cellulose gum, sodium hydroxide, sodium saccharin, cocamidopropyl betaine, hesperidin, sucralose, sodium chloride, cetylpyridinium chloride, sodium benzoate, CI 42090.	Germany
12	Kinder Karex™ toothpaste	Aqua, hydrogenated starch hydrolysate, hydrated silica, hydroxyapatite, xylitol, silica, cellulose gum, aroma, 1,2-hexanediol, caprylyl glycol, sodium methyl cocoyl taurate, sodium sulfate, sodium cocoyl glycinate, limonene	Germany
13	NanoXIM •CarePaste	(Synthetic nano-HA water-based suspension ingredient designed to be easily incorporated in oral care products.) hydroxyapatite (nano), Potassium Chloride, Microbial content, heavy metals.	Portugal
14	VITIS^®^ whitening toothpaste	Aqua, glycerin, sorbitol, silica, PVP, sodium lauryl sulphate, titanium dioxide, sodium monofluorophosphate, pentasodium triphosphate, perlite, sodium hexametaphosphate, xanthan gum, xylitol, tetrapotassium pyrophosphate, hydroxylapatite (nano), menthone glycerin acetal, sodium saccharin, potassium chloride, sodium methylparaben, potassium acesulfame, aroma.	Spain
15	INNOVA	Aqua, hydrated silica, hydrogenated starch hydrolysate, glycerin, PEG-8, hydroxyapatite, cellulose gum, aroma, sodium monofluorophosphate, cocamidopropyl betaine, sodium lauroyl sarcosinate, xylitol, propylene glycol, olaflur, *Stevia rebaudiana* leaf extract, anethole, citric acid, eucalyptol, o-cymen-5-ol, tocopheryl acetate, CI 77891, thymol, calcium lactate, *Vitis vinifera* seed extract, disodium EDTA, aspergillus/tannic acid ferment extract, glucose, inositol, sodium benzoate, potassium sorbate, limonene. Fluoride content—0,15% (1500 ppm).	Russian
16	MEGASONEX	Sorbitol, glycerin, hydroxyapatite (nano), water (aqua), silica, xylitol, tetrasodium pyrophosphate, sodium methyl cocoyl taurate, mica, titanium dioxide, sodium carboxymethylcellulose, citric acid, sodium saccharin, aroma	USA
17	WhiteWashLaboratories	Glycerin, aqua, calcium carbonate, xylitol, hydroxyapatite, potassium nitrate, hydrated silica, tetrasodium pyrophosphate, kaolin, sodium bicarbonate, pentasodium triphosphate, pvp, sodium monofluorophosphate, cocamidopropyl betaine, potassium chloride, xanthan gum, stevioside, *Mentha piperita* oil, bromelain, l-menthol, papain, urea peroxide, *Eucalyptus globulus* leaf oil, limonene.	UK

## Data Availability

The data presented in this study are available on request from the corresponding author.
